# Multidisciplinary Treatment of Hemifacial Microsomia: Several Clinical Cases

**DOI:** 10.3390/clinpract14060188

**Published:** 2024-11-08

**Authors:** Mónica Cano-Rosás, Juan Benito-Cano, Javier Benito-Cano, José María Diosdado-Cano, Pablo Benito-Duque, Adrián Curto

**Affiliations:** 1 Department of Surgery, Faculty of Medicine, University of Salamanca, Alfonso X El Sabio Avenue s/n, 37007 Salamanca, Spain; mcanorosas@usal.es; 2 Private Practice, 28034 Madrid, Spain; benitojuan587@gmail.com; 3 Helthcare NHS Foundation Trust, Birminghan B7 4BN, UK; javierbenitocano@gmail.com; 4 Newland Dental Care, Lincoln LN1 1YA, UK; jmdcodontology@outlook.es; 5 Servicio de Cirugía Plástica y Reparadora del Hospital Ramón y Cajal, 28034 Madrid, Spain; pbenitoduque@gmail.com

**Keywords:** auricular reconstruction, hemifacial microsomia, microtia

## Abstract

Hemifacial microsomia is the second most common congenital anomaly of the craniofacial region. Hemifacial microsomia is characterised by unilateral hypoplasia of the ear. Treatment of this condition depends on the severity of the lesion. The treatment of hemifacial microsomia must be carried out by a multidisciplinary group of professionals familiar with this pathology, including plastic surgeons, parapsychologists, orthodontists, and paediatricians. In hemifacial microsomia, microtia is usually accompanied by alterations of the middle ear. Since the ear develops embryonically from the first and second branchial arches, the facial areas that also develop from these embryonic origins are usually affected to a greater or lesser degree, including through hypoplasia of the jaw, maxilla, zygomatic bones, and temporal bone, among others. Although jaw hypoplasia is the most evident deformity in craniofacial microsomia, microtia is the alteration that often has the greatest aesthetic impact on patients. Alterations in dentition are also common, typically presenting as a cephalad inclination of the anterior occlusal plane of the maxilla and mandible on the affected side. This study aims to review the surgical approach and evaluate the results of a paediatric case of hemifacial microsomia. Hemifacial microsomia is present at birth, and successful reconstruction is essential for the correct integration of such infantile patients into society. Multiple facial asymmetries as well as neonatal onset are a challenge for reconstructive surgery, and the importance of multidisciplinary treatment in these patients must be emphasised.

## 1. Introduction

Craniofacial microsomia was first described in 1861 by Canton, who noted the association between ipsilateral mandibular and auricular anomalies. This disorder has since been referred to by several names, including first and second branchial arch syndrome, otomandibular dysostosis, Goldenhar syndrome, lateral facial dysplasia, hemifacial microsomia, and craniofacial microsomia [1,2].

Because this disorder can be bilateral in 5% to 30% of cases, it is advisable to use the terms “unilateral” and “bilateral” craniofacial microsomia. Craniofacial microsomia has an estimated occurrence of 1 in 5600 live births, making it the second most frequent craniofacial anomaly after cleft lip and palate [2,3,4]. However, the incidence varies according to different reports, ranging between 1 in 642 and 1 in 26,000 live births, suggesting that it depends on adequate diagnosis of the studied case series. In many cases, this syndrome goes unnoticed, and in some cases, not even the family realise that the affected person is showing signs of a hemifacial microsomia, especially since these dysplasias are characterised by their variability and different degrees of penetrance. Moreover, hemifacial microsomia is characterised by hypoplasia of the mandible, maxilla, and auricle, which manifests itself with greater or lesser intensity [2,3,4,5].

**Figure 1:**
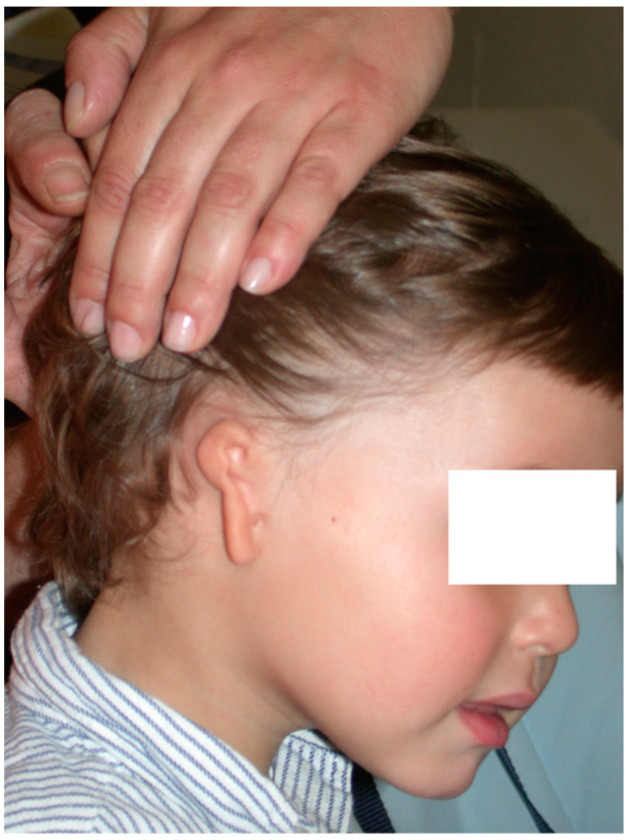
Severe auricular hypoplasia I.

**Figure 2:**
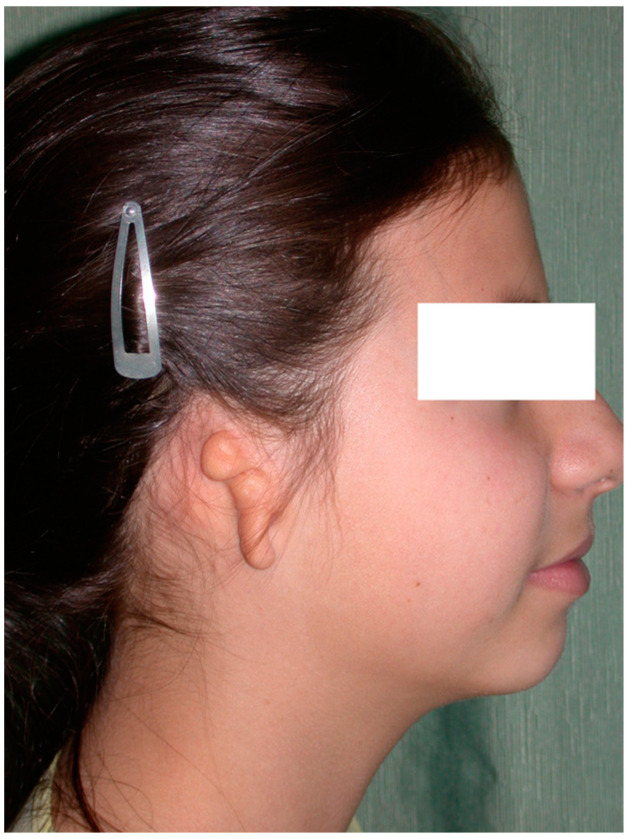
Severe auricular hypoplasia II.

In affected patients, the absence or atrophy of the auricle is sometimes the most visible alteration (Figure 1 and Figure 2), with a transcendental impact on the patient’s social experiences due to the serious distortion of their body image [6]. For this reason, we recommend informing the professionals attending to these patients about other associated pathologies, such as malocclusion (orthodontists), iris coloboma, and microphthalmia (ophthalmologists) and, less frequently, extra-craniofacial abnormalities of a renal, cardiac, or skeletal type (paediatricians) [6,7,8].

All structures dependent on the first and second branchial arches may be affected by craniofacial microsomia. The jaw is always affected to some degree (hypoplasia or agenesis of the condyle, alterations to the TMJ, three-dimensional reduction in the horizontal branch, etc.), while in the maxilla, there is no true volumetric deficiency, and its involvement is usually secondary to the inhibition of the mandible’s vertical growth. There may also be dysmorphogenesis of the tongue, and the VII cranial nerve may be affected, causing alterations to the muscles related to facial expression [9,10,11].

In some patients affected by hemifacial microsomia, it is necessary to plan orthognathic surgery treatment for mandibular reconstruction. In these patients who are candidates for mandibular reconstruction, it is essential to plan the surgery and the results of the surgery using cone beam computed tomography [12,13].

External or internal distractors also play an important role in these. Mandibular distraction osteogenesis is an important component in the treatment of patients with hemifacial microsomia. Because of the narrow surgical field of the intraoral approach, the accuracy of osteotomy largely depends on the surgeon´s experience [7,14,15,16].

The aim of this paper is to describe multidisciplinary treatment options for paediatric patients with hemifacial microsomia through the presentation of a clinical case.

## 2. Case Presentation

At present, autologous cartilage grafts are considered a necessary tool in auricle reconstruction. Such surgery should be delayed until the costal growth is adequate for use. Eight years old is considered a suitable age, since by this age, the ear has practically reached adult size and the child will be more cooperative [17,18,19,20].

**Figure 3:**
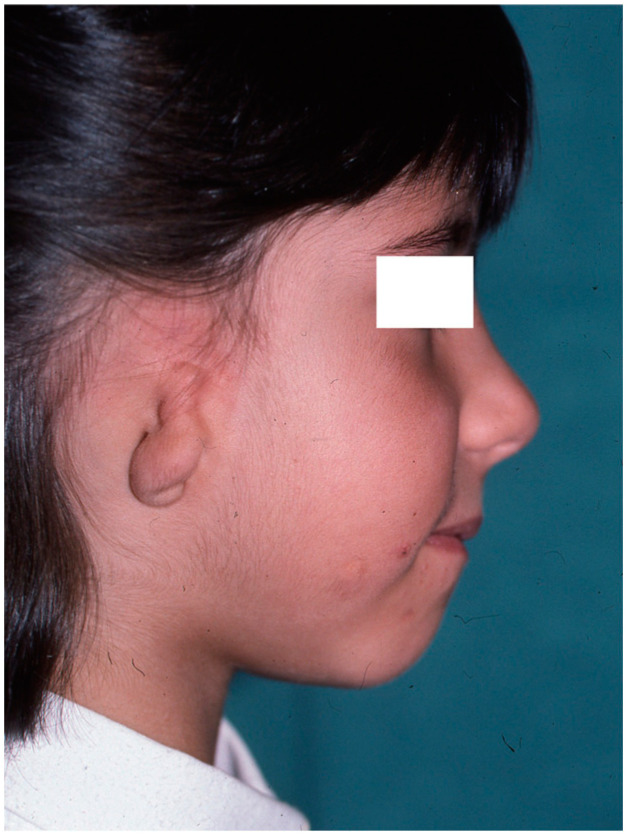
Preoperative calculation in the affected hemiface for the implantation of cartilage for auricular reconstruction.

**Figure 4:**
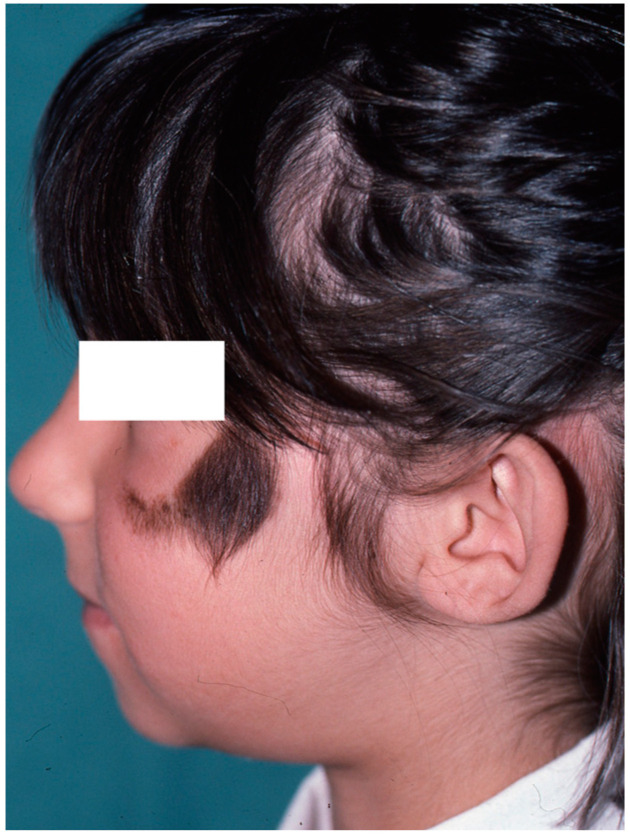
Image of healthy side taken as a reference for contralateral auricular reconstruction. Note the presence of a congenital nevus on the left cheek, which was treated by partial excision.

A good result is directly dependent on proper planning. The affected and healthy sides are photographed separately to calculate the location of the new ear and thus create a template to be used during the manufacture of the cartilaginous framework (Figure 3 and Figure 4).

**Figure 5:**
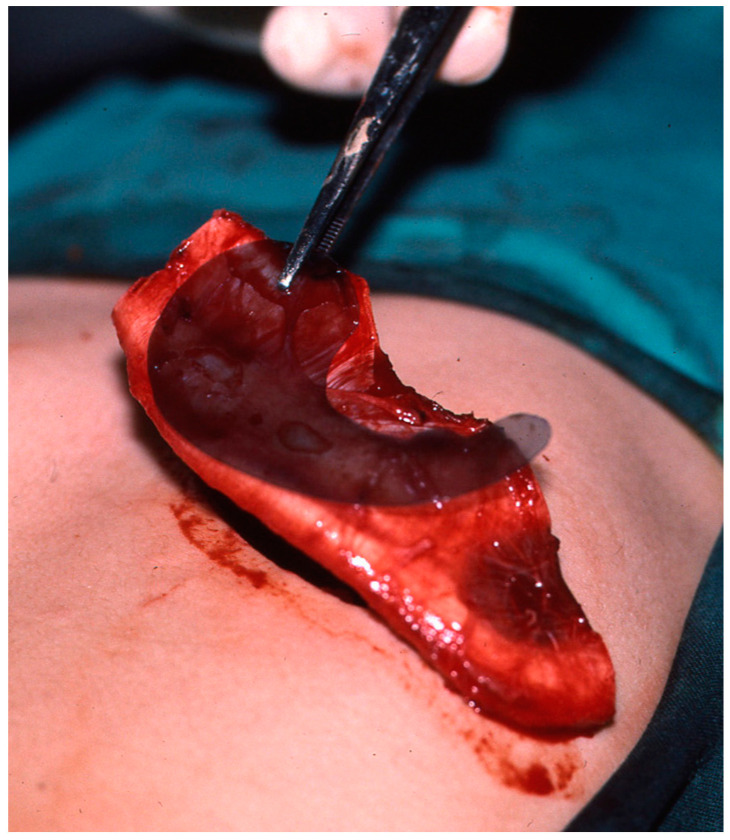
Application of a template for calculating the dimensions of the new cartilaginous framework after the costal cartilage has been extracted.

**Figure 6:**
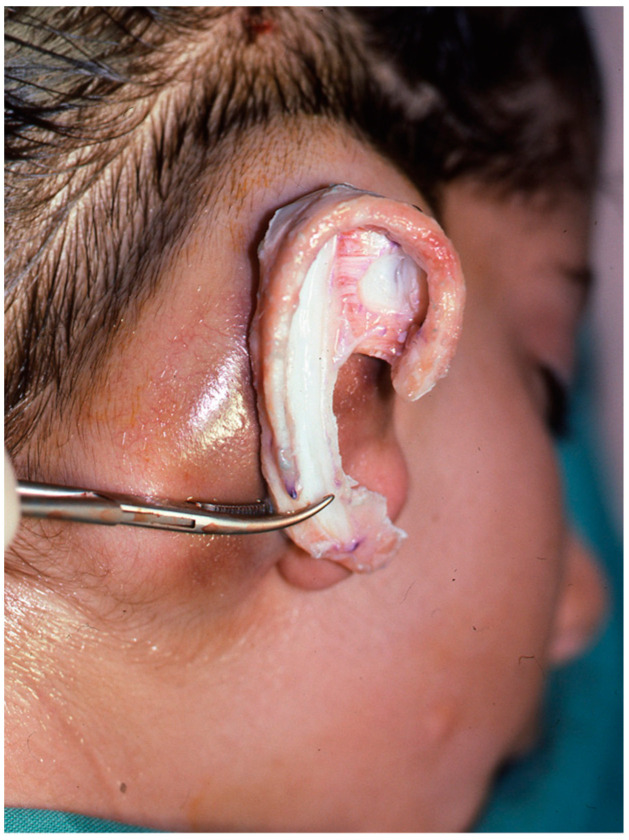
Manufactured cartilaginous structure prior to implantation in the subcutaneous space.

**Figure 7:**
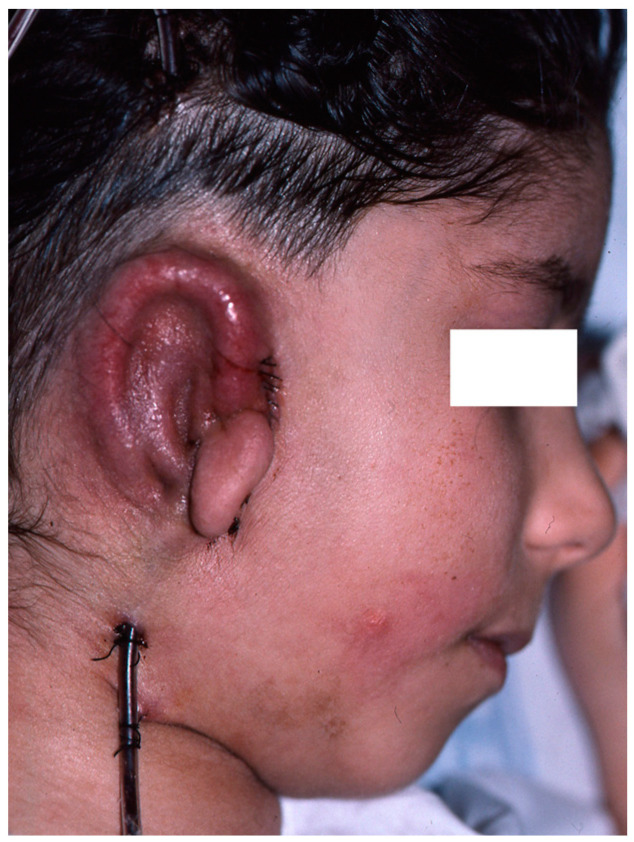
Cartilaginous structure introduced and vacuum system applied.

**Figure 8:**
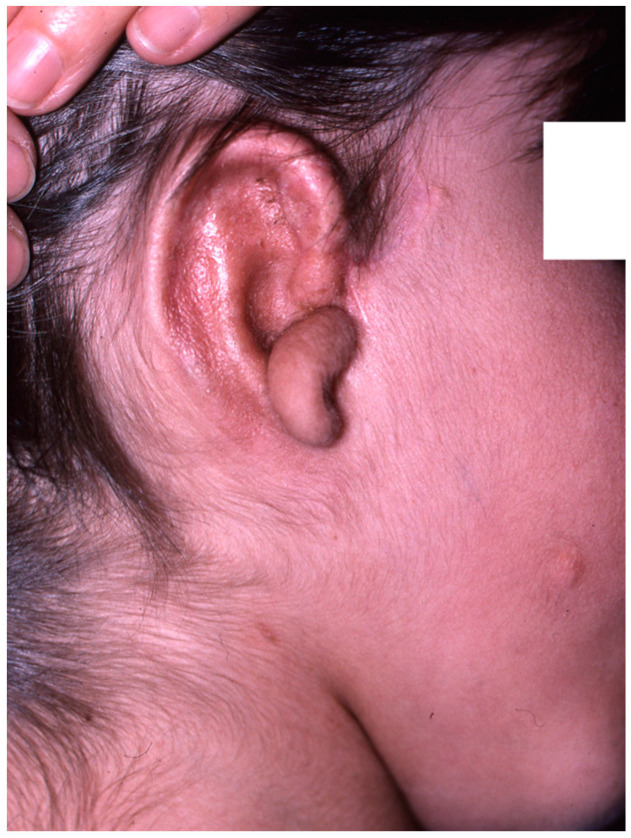
Ear lobe not yet rotated and without detaching the reconstruction of the mastoid.

**Figure 9:**
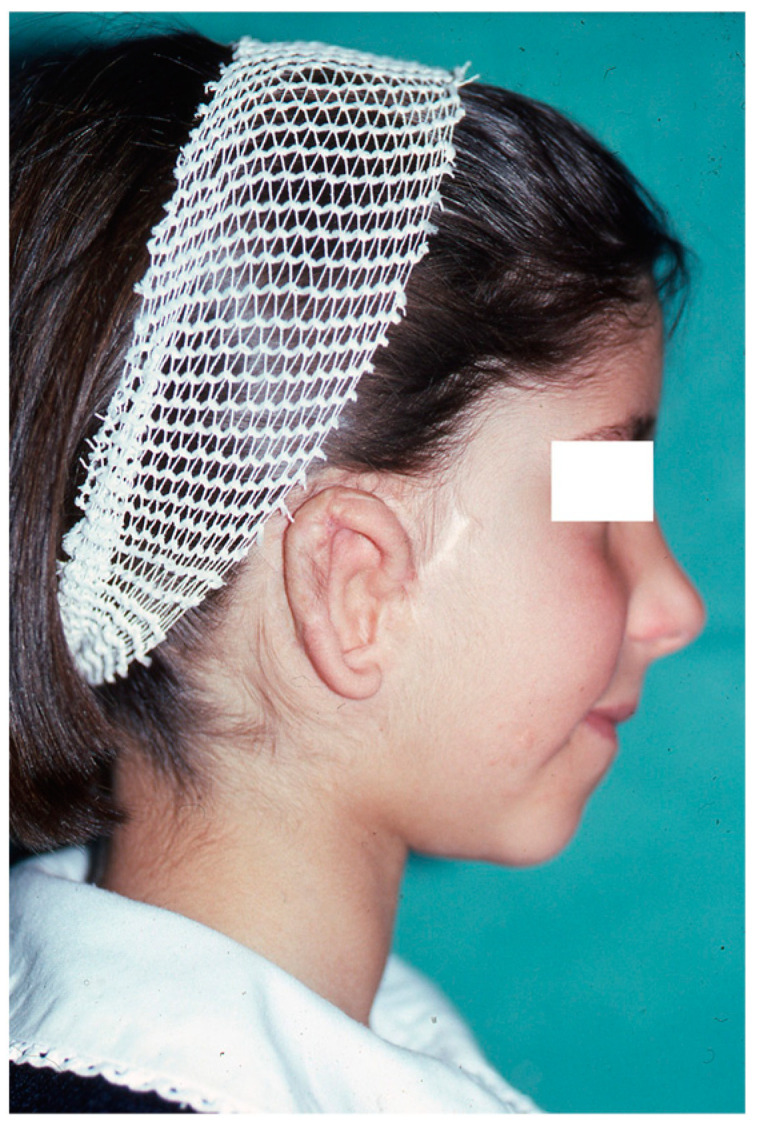
Final result in which an implantation base that is lower than the contralateral ear can be seen, in addition to slight deviation of the chin towards the affected side, absence of the natural “convexity” of the right cheek in relation to the healthy side, and a scar on the left cheek as a result of congenital nevus resectioning.

Reconstruction takes three or four interventions spaced 4–5 months apart. First, the costal cartilages are extracted from the side contralateral to the ear to be reconstructed, using the template that was made previously. With the cartilage removed, the new ear is manufactured (Figure 5 and Figure 6). It is then implanted in the subcutaneous space that is prepared during the same surgical intervention, and a vacuum system is applied that allows a perfect adaptation of the skin to the cartilage (Figure 7). In a later surgery, the lobe is mobilised (Figure 8). Finally, the tragus is manufactured, and the posterior surface of the ear is detached to form the posterior auricular sulcus by applying a skin graft (Figure 9) [15,16,18,19,21,22,23].

## 3. Discussion

Hemifacial microsomia sometimes presents with malformations of the zygomatic arch, skull base, and maxillary soft tissues. The most prominent features of patients with hemifacial microsomia are mandibular hypoplasia and microtia [1,3,4,5,7]. Because the ear develops from the tissues of the mandibular branchial arches and hyoid bone, it is common for many hemifacial microsomia patients to present with facial alterations in these anatomical structures, which share a common embryological origin. Evident facial asymmetry is observed in up to 35% of patients in some series. Moreover, 25% of patients have relatives with evidence of hemifacial microsomia to a greater or lesser degree, although all of them have bone involvement [1,24,25]. Therefore, hemifacial microsomia patients should undergo a comprehensive procedure focusing on the treatment of mandibular and auricle malformations.

Various etiopathogeneses have been considered. For example, there is a relationship between hemifacial microsomia and teratogenic agents—such as thalidomide, isotretinoin, clomiphene citrate, retinoic acid, and ethanol—as well as conditions that may contribute to developmental issues, such as gestational diabetes [1,26,27,28].

Likewise, a greater presence of these alterations has been found in first-degree relatives, although with highly variable penetrance. A fundamental characteristic of this syndrome is the diverse manifestation of clinical findings. Hemifacial microsomia is characterised by three main alterations: auricular, mandibular, and maxillary hypoplasia. Hypoplasia can affect the adjacent anatomical structures, including the facial nerve, middle ear, etc. [29,30].

**Figure 10:**
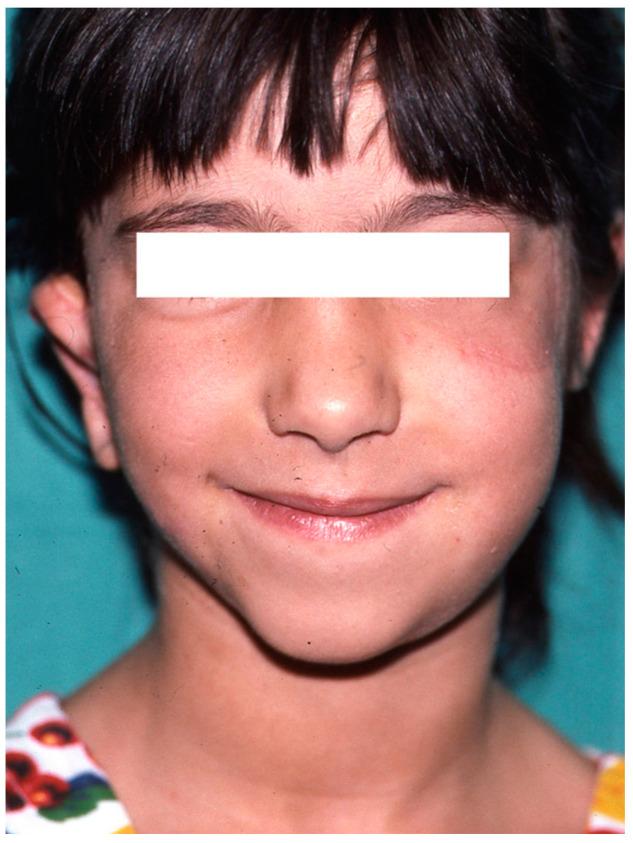
Frontal photograph showing deviation of the chin.

The most obvious deformity is mandibular hypoplasia on the affected side. The ascending ramus of the mandible is hypoplastic to a greater or lesser degree, causing the chin to deviate towards the affected side (Figure 10), with an increased angle of the mandible on the unaffected side. This alteration determines the inclination of the occlusal plane, higher on the affected side, with the corresponding alteration of the occlusion [31,32,33].

Various materials have been used for ear reconstruction, including polyethylene, nylon mesh, Marlex, Teflon, MEDPOR, and silicone [34], although only the use of autologous cartilage has stood the test of time in the implanted area. Costal cartilage is ideal for obtaining an adequately sized prosthesis, and it can be carved to obtain the right shape. Several studies have been carried out to develop facial reconstruction materials using tissue engineering techniques, but the development of these materials has not yet been extrapolated to daily clinical practice [35,36].

Patients with hemifacial microsomia have clinical features that pose difficulties for reconstruction, such as through auricular reconstruction, which is a complex procedure [29,34,36]. These patients often have severe mandibular deformities, resulting in the presence of a narrow pharyngeal space, which can cause difficulties during surgery [34]. A plastic surgeon, an otolaryngologist, and an orthodontist must be part of the multidisciplinary team that treats these patients. Because of the complex clinical manifestations of hemifacial microsomia, multidisciplinary cooperation is required to achieve better therapeutic effects in terms of function and aesthetics [37].

In patients with hemifacial microsomia, individualised treatment is currently recommended because of the different craniofacial malformations they present, and it is essential to focus treatment on the functional as well as the aesthetic level [38]. Patient assessments by the plastic surgeon and the orthodontist must be made early, and in the most severe cases of mandibular involvement, an assessment by an orthognathic surgeon is necessary [39].

Because of the malformations and oral alterations that these patients usually present, the involvement of dentists/orthodontists and oral surgeons in the treatment of orthognathic surgery is essential in the multidisciplinary management of these patients [12,40]. Orthodontists should plan the treatment of the dental malocclusions presented by these patients. It is important in clinical cases of hemifacial microsomia candidates for orthognathic surgery to have an interconsultation with the maxillofacial surgeon to coordinate surgical and orthodontic treatment [11,41].

## 4. Conclusions

Comprehensive treatment is imperative for paediatric hemifacial microsomia patients because of its complicated manifestations. Surgical treatment is recommended at around 8 years of age, based on different published studies. The success of surgical treatment of hemifacial microsomia is of fundamental importance in the psychological field of paediatric patients, and different specialties (plastic surgery, maxillofacial surgery, dentists, etc.) should be involved.

## Data Availability

No data in this paper reveal the patient’s identity. The data presented in this study are available upon request from the corresponding author.
